# Side effects from epidural analgesia in laboring women and risk of cesarean delivery

**DOI:** 10.1016/j.xagr.2023.100297

**Published:** 2023-12-12

**Authors:** Alessandro Ghidini, Kelly Vanasche, Alyssa Cacace, Marietta Cacace, Simona Fumagalli, Anna Locatelli

**Affiliations:** 1 Antenatal Testing Center, Inova Alexandria Hospital, Alexandria, VA (Dr Ghidini and Mses Vanasche, A Cacace and M Cacace); 4 Antenatal Testing Center, Inova Alexandria Hospital, Alexandria, VA, USA (Alyssa Cacace); 2 School of Medicine and Surgery, University of Milano Bicocca, Monza, Italy (Ms Fumagalli and Dr Locatelli); 3 Obstetrics, IRCCS San Gerardo dei Tintori, Monza, Italy (Ms Fumagalli and Dr Locatelli).

**Keywords:** cesarean delivery, epidural analgesia, fetal heart rate, hypotension, labor, obstetric analgesia

## Abstract

**BACKGROUND:**

Epidural analgesia may cause maternal hypotension and changes in the fetal heart rate. The implications of such side effects on the course of labor and delivery are incompletely understood.

**OBJECTIVE:**

This study aimed to assess whether the occurrence of maternal or fetal side effects associated with labor epidural analgesia increased the risk for cesarean delivery.

**STUDY DESIGN:**

This was a cohort study of all women who underwent epidural analgesia during labor for the period October 1, 2020 to December 31, 2020. Excluded were cases of multiples, fetal death, noncephalic presentation, and gestational age at birth <37.0 weeks. Maternal vital signs and fetal heart rate tracings for the 1 hour before and 1 hour after epidural analgesia was administered were reviewed. The occurrence of maternal hypotension, defined as a continuous variable and dichotomized into a decrease in maternal systolic blood pressure to <90 mm Hg or a drop in systolic blood pressure by >20% below the last value before epidural analgesia was administered, was related to changes in the fetal heart rate category. The principal outcome was cesarean delivery rate; binary logistic regression analysis was used to control for confounders, and mediation model analysis was used to quantify the extent to which significant variables participated in the causation pathway to cesarean delivery (SPSS version 28 was used for the analyses).

**RESULTS:**

A total of 439 women met the study criteria. Significant adverse reactions owing to epidural occurred in 184 of 439 women (41.9%) and included severe maternal hypotension in 159 of 439 participants (36.2%) and worsening fetal heart rate category in 50 of 439 participants (11.4%). The logistic regression analysis revealed that cervical dilation at epidural (*P*=.03), the duration of labor after epidural (*P*<.001), and worsening fetal heart rate category within 60 minutes of epidural administration (*P*=.01) were independently associated with recourse to cesarean delivery. The mediation analysis showed that both cervical dilatation at epidural administration and worsening fetal heart rate category had significant direct and indirect effects in the pathway to cesarean delivery.

**CONCLUSION:**

Worsening fetal heart rate category related to labor epidural independently increased the risk for cesarean delivery.

**AJOG Global Reports at a Glance**

**Why was this study conducted?**

Maternal hypotension related to epidural analgesia before elective cesarean delivery is an independent risk factor for neonatal acidemia and related complications. Epidural analgesia in laboring women can lead to maternal hypotension and changes in the fetal heart rate. The significance of such side effects on the outcome of labor and delivery is little studied.

**Key findings**

Maternal hypotension related to labor epidural is associated with fetal heart rate decelerations. Worsening fetal heart rate category within 60 minutes of an epidural is independently associated with recourse to cesarean delivery.

**What does this add to what is known?**

Our findings add to the controversial evidence on the association between labor epidural and cesarean delivery. An increased risk for cesarean delivery occurs only among women with fetal side effects caused by labor epidural. Protocols of care for labor epidural that minimize the risk for analgesia-related fetal heart rate changes may also reduce the risk for cesarean delivery.

## Introduction

The perinatal consequences of epidural analgesia before cesarean delivery (CD) have come under scrutiny. Although labor epidural has not been associated with increased risks for cesarean delivery or 5-minute Apgar scores <7, the interrelationship between maternal (namely hypotension) and fetal side effects (namely fetal heart rate changes) and labor epidural has not been adequately evaluated. With scheduled CD, the severity of maternal hypotension after spinal analgesia is independently associated with a decrease in umbilical artery pH.^1^, ^2^, ^3^ A decrease in pH reaching the threshold of fetal acidemia (ie, pH <7.20) is associated with a significant increase in neonatal morbidity.^2^

It is less clear if labor epidural have similar side effects that impact fetal well-being. A retrospective study documented the occurrence of fetal bradycardia in 11% of the cases analyzed with a peak incidence of onset of bradycardia occurring at 5 to 20 minutes after administration of epidural analgesia, but it continued throughout the 60-minute period after epidural.^4^ The transient fetal heart rate (FHR) changes did not impact neonatal outcomes. However, the authors did not correlate the FHR changes with the maternal blood pressure (BP) changes or with mode of delivery. A French study found anomalies in FHR during the hour following administration of labor epidural in 14.1% of cases; the CD rate was significantly higher (21.4% vs 9.6%; *P*<.002) and the incidence rate of umbilical artery pH <7.20 was higher (13.6% vs 6.5%; *P*<.002) among cases with FHR changes than among cases without.^5^ However, a multivariate analysis was not used to control for confounders.

The physiological mechanisms that lead to FHR anomalies immediately following labor epidural are not fully elucidated. Labor analgesia can be accomplished with a variety of local anesthetic agents, the use of adjuvant analgesic drugs with a local anesthetic, and different techniques of initiation and of maintenance of epidural analgesia, all of which may contribute to the observed side effects on the FHR. Possible mediating factors include maternal hypotension after epidural analgesia, which lead to decreased perfusion of the placenta; rapid onset of epidural analgesia may lead to a decrease in the plasma levels of maternal catecholamines, which has a beta-mimetic effect and leads to uterine hypertonus^6^, ^7^, ^8^; techniques of initiation of analgesia with higher rates of fetal bradycardia reported after combined intrathecal and epidural opioids^9^^,^^10^; intrathecal administration of adjuvant opioids together with the anesthetic mixture^10^^,^^11^; and maternal physiological characteristics^12^ and fetal vulnerability.^13^

The aim of this study was to better clarify the interdependence between fetal and maternal side effects and labor epidural and the possible effects on the course of labor and risk for CD.

## Materials and Methods

In this retrospective cohort study, we included all pregnant women who received epidural analgesia during labor for the period October 1, 2020 to December 31, 2020, at the Inova Alexandria Hospital, Alexandria, VA. A sample size of convenience was adopted to analyze the side effect of epidural analgesia on laboring women. Excluded were cases with twins or higher-order multiples, fetal death, noncephalic presentation, or gestational age <37.0 weeks at birth.

All women in the study were administered 1 L of Ringers lactate before epidural analgesia; only the epidural technique was used for labor analgesia (ie, spinal epidural was not used). There was not a single concentration and dose of anesthetic used for epidural analgesia. All laboring women with epidural analgesia had sequential compression devices routinely installed.

For all women in the study, the maternal vital signs were collected. Per institution policy, maternal vital signs were assessed every 5 minutes for 15 minutes after epidural placement, then every 15 minutes for 30 minutes, and then every 30 minutes. The last maternal BP recorded before epidural analgesia and the lowest maternal BP reading during the hour after completed administration of epidural analgesia were recorded. We calculated the changes in systolic BP from before epidural to the nadir after the epidural (in mm Hg) and the percentage of change in systolic BP (calculated as last systolic BP before epidural minus systolic BP nadir after epidural divided by systolic BP before epidural). Maternal hypotension was defined according to the institutional labor and delivery policy for care of epidural analgesia in laboring patient as a decrease in maternal systolic blood pressure (BP) to <90 mm Hg or a drop in systolic BP of >20% below the last value before administration of epidural analgesia. Per institutional policy, in the event of such a degree of hypotension, symptomatic maternal hypotension, or changes in FHR tracing after labor epidural, appropriate nursing responses were initiated, including increasing the intravenous (IV) fluid rate, administration of oxygen, left side lying position, and administration of 5 to 10 mg of ephedrine IV. Persistence of epidural-related side effects despite the above measures prompted an alert to the anesthesiology and obstetrical care providers. The FHR tracings during the hour before epidural administration and the hour after epidural adminsitration were evaluated by a reviewer (A.G.) blinded to the labor outcome and were categorized according to the American College of Obstetrics and Gynecology (ACOG) classification.^14^ The reviewer held a recent (<2 years) certification in advanced electronic fetal monitoring obtained through the General Electric Healthcare Learning System. In addition, the incidence of severe decelerations in FHR tracings was recorded, including prolonged decelerations of ≥2 minutes but <10 minutes in duration, bradycardias lasting ≥10 minutes, recurrent late decelerations, and recurrent severe variable decelerations.^6^^,^^7^ In the presence of severe decelerations, the duration of decelerations was quantified by calculating the total time (in seconds) that the FHR was below baseline.^15^

This study was approved by the institutional review board committee (U21-04-4428 approved on June 16, 2021).

### Statistical analysis

Two exposures could be related to the outcome, namely maternal hypotension and FHR worsening ACOG category. The interrelationship between 2 potential primary exposures, which has been poorly studied, made it difficult to make an a priori hypothesis about a single primary exposure. Binary logistic regression analysis was used to evaluate the association between maternal hypotension and FHR changes and the association between side effects of labor epidural and recourse to CD. To explore whether a time relationship existed between significant adverse side effects to the labor epidural and CD, linear regression analysis was used with the interval to delivery after completion of epidural analgesia as a dependent variable.

Mediation analysis was performed to explore the roles of explanatory variables as predictors and possible mediators of CD. The mediation analysis consisted of the traditional 4 steps for testing mediation.^16^ The mediation model was applied according to the following strategy. We selected variables significantly and independently associated with CD in the multivariate logistic regression analysis. We explored the possible impact that cervical dilatation at epidural administration, mediated by the duration of labor after epidural, may have on CD. The same approach was used to explore a possible mediation between cervical dilatation at epidural and worsening FHR category on CD. A path diagram of the single mediator model was used to represent the relationship between variables.

## Results

During the study period, 501 women received epidural analgesia during labor. From this data set, we excluded 10 with twins or higher-order multiples, 1 with fetal death, and 51 with gestational age <37.0 weeks at birth, leaving 439 laboring women who met the study criteria. Women self-identified as White in 42.6% of cases, Black in 21.3% of cases, Asian in 7.5% of cases, other in 0.3% of cases, and the remainder (28.3%) did not associate with any race.

Significant associations were noted between the difference in systolic BP from before to after epidural and changes in the FHR category (*P*=.015) and the occurrence of severe FHR decelerations (*P*=.004). Significant associations were also noted between the percentage change in systolic BP and worsening FHR category (*P*=.021) and the occurrence of severe FHR decelerations (*P*=.003) (Table 1).

**Table 1:** Correlation between maternal BP changes and FHR variables in the absence of severe maternal hypotension (*P* values)

BP parameters	Worsening in FHR category	Severe FHR decelerations	Duration of FHR below baseline
Delta systolic BP	.015	.004	.008
Percentage change in systolic BP	.021	.003	.006
Maternal hypotension	.32	.03	.01

*BP*, blood pressure; *FHR*, fetal heart rate.

**Figure 1:**
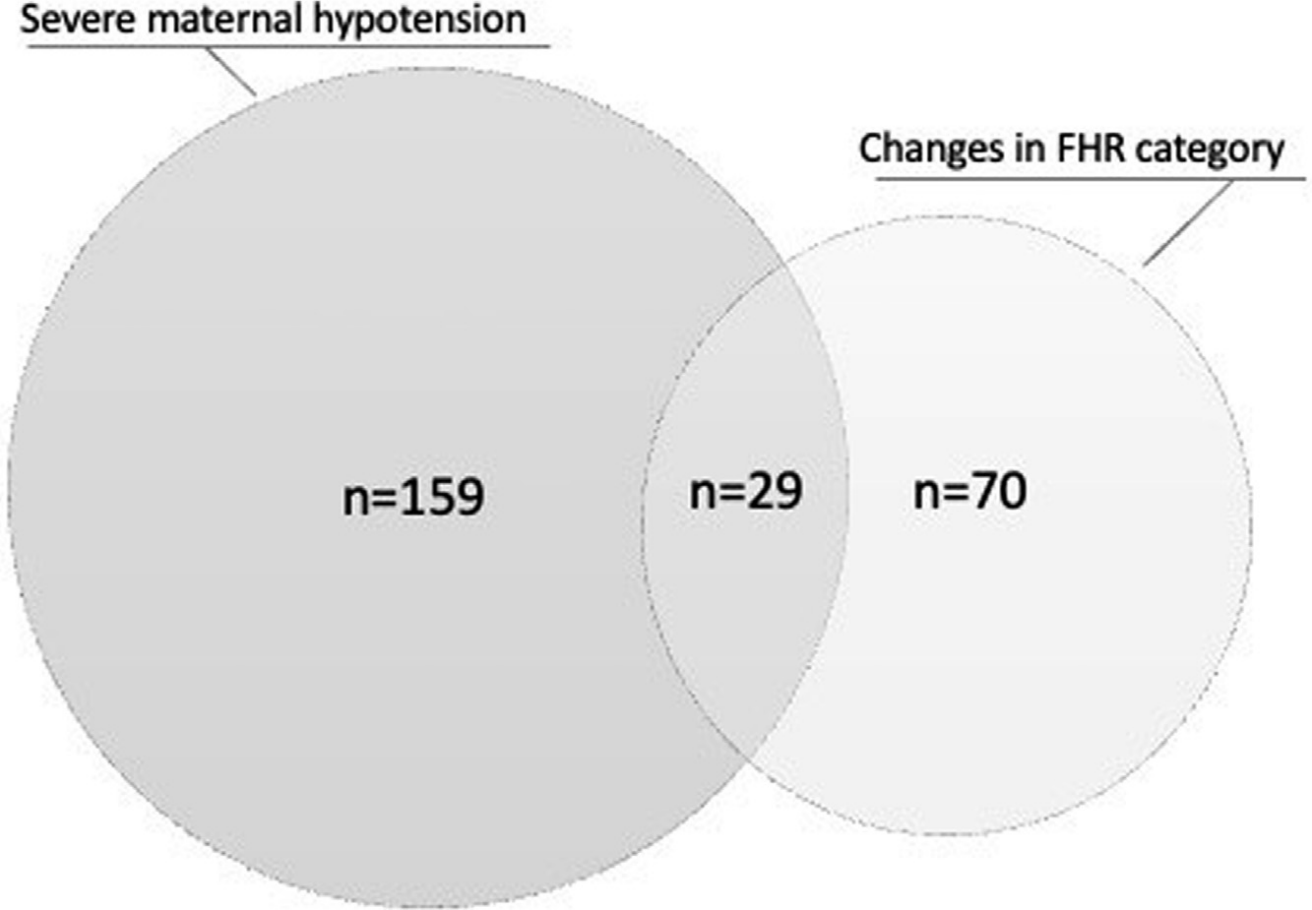
Severe maternal hypotension and changes in FHR category *FHR*, fetal heart rate.

Maternal hypotension, defined according to the existing policy at our institution for laboring epidural (ie, decrease in systolic BP to <90 mm Hg or by >20% below the pre-epidural BP), occurred in 159 cases (36%) of which 24 had a decrease in systolic BP to <90 mm Hg. Maternal hypotension was associated with the incidence of severe FHR decelerations (25% or 15.8% vs 25% or 8.9%; *P*=.04) but not with changes in FHR category (29% or 18.4% vs 41% or 14.6% in the absence of severe hypotension; *P*=.34). Duration of FHR below the baseline in the presence of severe decelerations was not associated with a difference in the systolic BP from before to after the epidural (*P*=.26), with the percentage change in the systolic BP (*P*=.27), or with the occurrence of hypotension (mean ± SE, 378±59 seconds with hypotension vs 248±40 seconds without hypotension; *P*=.067). In the absence of maternal hypotension, neither a difference in systolic BP (from before to after epidural administration) nor a percentage change in the systolic BP was significantly associated with changes in the FHR category (*P*=.74 and *P*=.84, respectively), with the occurrence of severe FHR decelerations (*P*=.09 and *P*=.09), or with the duration of FHR below baseline after epidural in the presence of severe decelerations (*P*=.75 and *P*=.73). Of interest, among the 159 cases with maternal hypotension, 29 (18.2%) had changes in the FHR category and 25 (17.7%) had severe FHR decelerations. However, 41 cases (14.6%) with changes in the FHR category and 25 cases (8.9%) with severe FHR decelerations occurred without maternal severe hypotension (Figure 1).

Ghidini. Epidural analgesia in labor and risk of cesarean delivery. Am J Obstet Gynecol Glob Rep 2023.

**Figure 2:**
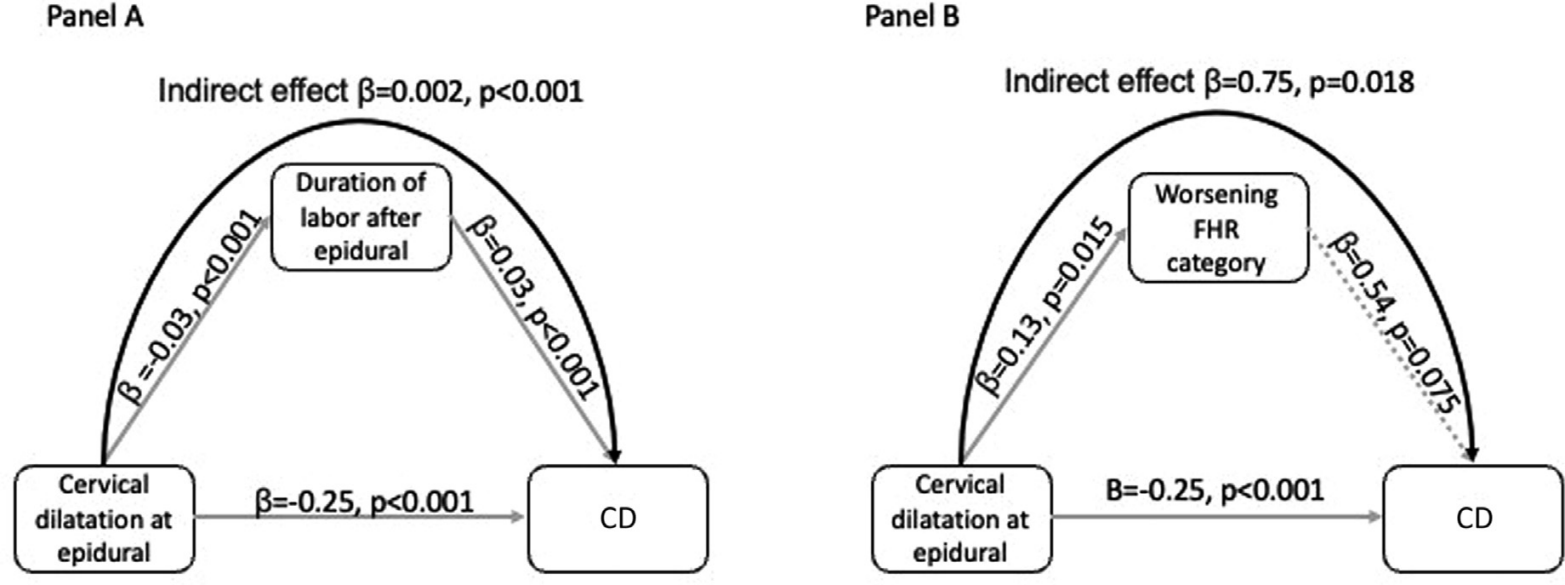
Mediation analysis model Mediation analysis model with cesarean delivery (CD) as outcome, including cervical dilatation at epidural and duration of labor as mediators (**Panel A**). Mediation analysis model with CD as outcome, including cervical dilatation at epidural and worsening FHR category as mediators (**Panel B**). *Grey line*, direct effect; *black line*, indirect effect. *CS*, cesarean section; *FHR*, fetal heart rate.

To evaluate if significant side effects of labor epidural were associated with the incidence of CD, we compared maternal obstetrical, labor, and epidural-related variables with the mode of delivery (Table 2). Cases that required CD had a greater body mass index, less cervical dilation at the time of epidural placement, a longer duration of labor after epidural, a higher incidence of severe adverse reactions to epidural requiring vasopressors, and a greater birth weight. Only one case in our cohort had an Apgar score of <7 at 5 minutes. When we performed the multivariate logistic regression analysis, cervical dilation at epidural placement (adjusted odds ratio [aOR], 0.86; 95% confidence interval [CI], 0.75–0.98; *P*=.03), duration of labor with epidural (aOR, 1.002; 95% CI, 1.001–1.003; *P*<.001), and worsening FHR category (aOR, 2.3; 95% CI, 1.2–4.5; *P*=.011) were significantly and independently associated with CD (Table 3). Of interest, in the subgroup of the 85 cases that required CD, only worsening FHR category with epidural (OR, 3.8; 95% CI, 1.29–11.06; *P*=.015) was associated with an increased risk for CD for nonreassuring fetal status. To better explore if worsening FHR category was related to CD, we performed a mediation analysis using CD as the outcome (Figure 2). Both cervical dilatation at epidural and worsening FHR category had significant direct and indirect effects in the causal pathway to CD. The interval to delivery after labor epidural analgesia was associated with maternal age (*P*=.007), body mass index (*P*<.001), cervical dilation at epidural (*P*<.001), severe deceleration (*P*=.021), and the incidence of severe adverse reactions to epidural (*P*=.019), cesarean delivery (CD) (*P*<.001), and CD for nonreassuring FHR (*P*=.009). The linear regression demonstrated that only cervical dilation at epidural (*P*<.001) and CD (*P*<.001) were independently related to the interval to delivery after epidural, whereas adverse reactions to epidural were not (*P*=.22), suggesting that adverse reactions to epidural analgesia did not lead to a shorter interval to delivery.

**Table 2:** Demographic, obstetrical, and blood pressure parameters and FHR findings in relation to recourse to cesarean delivery (CD)

Parameter	Cesarean delivery (n=85)	Vaginal delivery (n=354)	Coefficient	*P* value
Age (y)	31.0 (0.5)	31.6 (0.3)	0.9	.34
BMI	31.4 (0.7)	29.7 (0.3)	5.7	.017
Nulliparity	44 (51.8%)	165 (46.6%)	0.041	.40
Gestational age (wk)	39.2 (1.05)	39 (1.03)	2.01	.10
Cervical dilatation at initiation of epidural (cm)	3.6 (0.2)	4.7 (0.1)	17.4	<.001
Duration of labor after epidural (min)	645 (33.4)	391 (14.8)	54.8	<.001
History of cesarean delivery	4 (4.7%)	8 (2.3%)	0.06	.26
Induced labor	45 (52.9%)	167 (47.2%)	0.047	.34
Birth weight (g)	3478 (48.3)	3347 (22.2)	6.5	.02
Birth weight <10th percentile^d^	6 (6.9%)	25(7%)	0.031	.66
Significant side effects of epidural	38 (44.7%)	146 (41.2%)	0.028	.60
Need for vasopressor	39 (45.8%)	105 (29.6%)	0.14	.007
FHR changes after labor epidural^a^				
Worsening FHR category	19 (22.3%)	51 (14.4%)	0.086	.087
Severe FHR decelerations	10 (11.8%)	40(11.3%)	0.006	.90
Duration FHR below baseline in the presence of severe decelerations	499 (104)	235 (38)	0.21	.27
Maternal BP changes with epidural^a^				
Delta systolic BP (mm Hg)^b^	18.9 (1.8)	19.3 (0.8)	0.04	.84
Percentage decrease in systolic BP^b^	14.1 (1.3)	14.5 (0.6)	0.09	.76
Maternal hypotension^c^	35 (41.1%)	124 (35.0%)	0.05	.32

The data are presented as number (percentage or standard error).

*BMI,* body mass index; *BP*, blood pressure; *FHR*, fetal heart rate.

a Assessed during the hour before and the hour after epidural placement

b Between the last BP recorded before epidural and nadir within 1 hour post epidural

c Defined as a systolic blood pressure<90 mm Hg or a drop in SBP >20% below the last value before epidural anesthesia

d According to normative data by Duryea et al 2014.^17^

**Table 3:** Results of logistic regression analysis for predictors of cesarean delivery

Predictors	Odds ratio	95% CI	*P* value
BMI	1.03	0.98–1.07	.24
Cervical dilatation at epidural	0.86	0.75–0.98	.03
Duration of labor after epidural	1.002	1.001–1.003	<.001
Worsening FHR category	2.3	1.2–4.5	.011
Birth weight	1.000	1.000–1.001	.165

*BMI*, body mass index; *CI*, confidence interval; *FHR*, Fetal heart rate.

Ghidini. Epidural analgesia in labor and risk of cesarean delivery. Am J Obstet Gynecol Glob Rep 2023.

## Comment

### Principal findings

We have found that epidural analgesia in labor may lead to changes in both the maternal BP and FHR; these 2 phenomena are significantly related with one another. Of interest, changes in the FHR category but not in the maternal BP seemed to be independently associated with an increased risk for CD. Such an association was independent of the maternal and fetal characteristics analyzed, and it persisted even after accounting for factors during labor (eg, cervical dilation at epidural placement).

The current recommendations by the Society of Obstetric Anesthesia include monitoring the FHR before and after the administration of epidural analgesia for labor because of changes in the FHR patterns.^18^ However, such FHR changes have not been considered to be clinical relevant because they are transient and are not associated with worse neonatal outcomes.^4^ Our findings confirm that Apgar scores and the rates of neonatal intensive care unit admission are not associated with the side effects of labor epidural, however, in our cohort, such good outcomes came at the price of an increased risk for CD once a worsening FHR category was noted during the 60 minutes after epidural analgesia. If our findings are confirmed by other investigators, more attention should be paid to the occurrence and prevention of fetal side effects caused by epidural analgesia.

### Results in the context of what is known

A systematic review of randomized clinical trials has demonstrated no increased risk for CD among studies that compared epidural analgesia with opioids during labor (risk ratio [RR], 1.07; 95% CI, 0.96–1.18), among those that compared epidural analgesia with placebo (RR, 0.46; 95% CI, 0.23–0.90), or those that compared epidural analgesia with continuous support (RR, 1.21; 95% CI, 0.91–1.62). Of note, changes in FHR were not analyzed.^19^ However, the evidence that shows a lack of an effect of labor epidural on the risk for CD is currently being challenged. A critical reanalysis of the randomized clinical trials included in the Cochrane database that compared labor epidural with opioid analgesia that showed that labor epidural had no effect on CD rate lack external validity in much of North American practices because of the marked effect of the dose of oxytocin on the risk for CD.^20^ In the study of Wong et al,^21^ intrathecal analgesia was associated with a significant increase in prolonged decelerations, late decelerations, and nonreassuring fetal heart rate changes (defined as those that would lead to an obstetrical intervention). However, the study did not assess the rates of maternal hypotension and thus could not evaluate any association between maternal and fetal side effects.^21^ Large cohort studies have also showed surprising findings. A study based on the National Slovenian Perinatal database found that among 68,790 nulliparous term women with singleton fetuses in cephalic presentation in spontaneous labor, epidural analgesia was associated with a significantly higher CD rate when compared with no epidural (13.3% vs 10.1%; *P*=.003).^22^ Similar findings were reported in a prospective Danish cohort study of 2721 term nulliparous women with spontaneous labor and a singleton fetus in cephalic presentation that showed that women with epidural analgesia had a higher risk for emergency CD after adjusting for multiple confounders (aOR, 5.8; 95% CI, 4.1–8.1).^23^ A 10-year retrospective National Dutch cohort study found a positive association between epidural analgesia and CD in both nulliparous (aOR, 1.99; 95% CI, 1.95–2.03) and multiparous women (aOR, 2.86; 95% CI, 2.76–2.97).^24^ Two large studies that used propensity score analysis found that epidural analgesia in labor was associated with an increased risk for CD when compared with nonepidural analgesia (RR, 2.5; 95% CI, 2.5–2.6;^25^ and OR, 1.6; 95% CI, 1.5–1.7).^26^ Finally, a recent study that used a nationwide birth registry of 380,935 singleton pregnancies at ≥36.0 weeks’ gestation showed a significant increased risk for emergency delivery owing to fetal compromise among women with epidural analgesia, irrespective of parity.^13^ The discrepant findings between randomized clinical trials and cohort studies may be because of the difficulty randomized trials, typically of a small size, face in detecting an increase in CD when limited to a subset of women with fetal side effects caused by labor epidural, which may become more evident with the larger numbers typically included in cohort studies. Moreover, direct conclusions from randomized clinical trials can be applied to the same intervention in the same study population with reasonable certainty; however, the external validity of the results is not guaranteed when extrapolating the findings to similar, but not identical, interventions or to other patient populations. Patients who agree to participate, even in well-designed clinical trials, may be, and often are, different from the general population. Thus, it is important to question how the findings from randomized clinical trials can be applied to and translate into every-day clinical practice. Studies that evaluate the translation of clinical trial results into the real world (not the controlled environment of a trial) are valuable in shaping our understanding of a study's generalizability and providing us with additional evidence to better inform our clinical decision-making and patient counseling.

Changes in labor anesthesia techniques and protocols over the past decades have been driven mainly by a desire to optimize maternal analgesia while avoiding an impact on the rates of operative deliveries. Our attempt to tease out the relationship between maternal and fetal side effects and labor epidural may lead to an increased awareness of these issues.

Our granular examination of FHR tracings before and after epidural analgesia and the ability to control for several potential confounders support an independent association between the side effects of epidural analgesia and an increased risk for CD, particularly in the presence of a worsening FHR category. Large studies are needed to deepen the understanding of the relationship between cervical dilatation at epidural and the epidural-related FHR changes associated with recourse to CD.

A systematic review that included 9 studies (total number of 15,752 women) showed no clinically meaningful difference in the risk for CD with early initiation vs late initiation of epidural analgesia for labor (RR, 1.02; 95% CI, 0.96–1.08).^27^ Once again, such studies may not have adequate statistical power to detect a significant increase in the risk for CD in the subset of patients with epidural-related changes in FHR.

### Clinical implications

Our findings highlight the role that anesthesiology interventions may play in the causal pathway leading to CD. Differences in primary CD rates across hospitals have been documented to span from 9% to 37%,^28^ and such variation was not explained by some facilities having more higher- or lower-risk patients than others. Traditionally, obstetrical care providers have been considered important drivers of the observed variation.^29^^,^^30^ Our findings, however, indicate the relevance of the protocols of care adopted by analgesia providers in the management of epidural analgesia during labor. Of particular interest is the role played by FHR changes in the causal pathway; traditionally, FHR changes are deemed secondary to maternal hypotension. Our results showed that the majority of FHR changes were not associated with severe maternal hypotension, yet they may drive a higher recourse to CD. Different fetal vulnerabilities to even mild degrees of maternal hypotension may explain the findings. The vasopressor medication used at our institution (ephedrine) does not seem to be responsible for the increased risk for CD at the doses administered to laboring women after epidural analgesia. This reassuring observation is in line with previous small studies that showed that a continuous IV infusion of ephedrine for 60 minutes after epidural analgesia was associated with a significantly higher mean BP and a lower rate of major FHR changes when compared with no ephedrine.^31^ Prophylactic intramuscular ephedrine at the time of combined spinal epidural in labor has also been shown to reduce the incidence of analgesia-related maternal hypotension and the incidence and frequency of late decelerations.^32^

### Research implications

Although vasopressors may rapidly correct maternal symptoms caused by hypotension related to epidural analgesia during labor, there is a paucity of evidence on the benefit of vasopressors in improving fetal status as manifested by immediate FHR changes following epidural analgesia during labor. The findings of this study were noted despite liberal use of ephedrine for maternal and fetal side effects of labor epidural. Studies are needed to establish whether therapeutic administration of vasopressors in the presence of epidural-related FHR changes attenuate the recourse of CD in comparison with traditional resuscitative interventions. Indeed, a recent study has shown that the majority of category II FHR patterns during labor improve to category I within 60 minutes of resuscitative interventions (mainly oxygen administration and IV fluids).^33^ If therapeutic vasopressors are effective in reducing recourse to CD, studies are needed to investigate whether ephedrine or phenylephrine is better, because it already has been studied for epidural analgesia before elective CD.

More research is also needed to evaluate if certain fetuses, such as those with growth restriction, are more vulnerable to the hypotensive effects of labor epidural and respond with FHR decelerations and worsening FHR category.

### Strengths and limitations

The accurate review of changes in maternal and fetal variables around the time of epidural placement, although it is time consuming and requires specific expertise for interpretation, was a strength of our study because it provided objective evidence of the occurrence and severity of side effects of epidural analgesia, strengthening the robustness of the results.

A limitation of our study is the lack of standardization in the kits and protocols used for labor epidural in our setting. Moreover, we did not evaluate the uterine contractile activity or the use of uterotonics for the induction and augmentation of labor. However, protocols of care for cervical ripening and induction of labor were uniformly applied during the study period and were not different among patients who received epidural analgesia. Another limitation is the relatively small number of patients with obstetrical pathologies (eg, preeclampsia or small for gestational age), which precluded an analysis of the possible causal role of such vulnerabilities on the risk for epidural side effects. Similarly, the small number of CDs for fetal intolerance to labor did not allow a subanalysis to determine if a specific type of CD was affected by the side effects of labor epidural.

### Conclusion

This study has shown an independent association between the side effects of epidural analgesia during labor, particularly worsening FHR category, and the risk for CD. Because lowering the CD rate is considered a worthwhile goal, comparisons of different protocols of care for epidural analgesia during labor may identify the optimal protocol to minimize such side effects.

## CRediT authorship contribution statement

**Alessandro Ghidini:** Conceptualization, Formal analysis, Methodology, Writing – review & editing. **Kelly Vanasche:** Conceptualization, Data curation. **Alyssa Cacace:** Conceptualization, Data curation, Formal analysis. **Marietta Cacace:** Data curation, Formal analysis, Writing – original draft. **Simona Fumagalli:** Formal analysis, Methodology, Writing – original draft. **Anna Locatelli:** Conceptualization, Formal analysis, Methodology, Writing – original draft, Writing – review & editing.

## References

[1] Rimsza RR, Perez WM, Babbar S, O'Brien M, Vricella LK (2019). Time from neuraxial anesthesia placement to delivery is inversely proportional to umbilical arterial cord pH at scheduled cesarean delivery. Am J Obstet Gynecol.

[2] Bligard KH, Cameo T, McCallum KN (2022). The association of fetal acidemia with adverse neonatal outcomes at time of scheduled cesarean delivery. Am J Obstet Gynecol.

[3] Hassanin AS, El-Shahawy HF, Hussain SH (2022). Impact of interval between induction of spinal anesthesia to delivery on umbilical arterial cord pH of neonates delivered by elective cesarean section. BMC Pregnancy Childbirth.

[4] Stavrou C, Hofmeyr GJ, Boezaart AP (1990). Prolonged fetal bradycardia during epidural analgesia. Incidence, timing and significance. S Afr Med J.

[5] Korb D, Bonnin M, Michel J, Oury JF, Sibony O (2013). Analysis of fetal heart rate abnormalities occurring within one hour after laying of epidural analgesia. J Gynecol Obstet Biol Reprod (Paris).

[6] Capogna G (2001). Effect of epidural analgesia on the fetal heart rate. Eur J Obstet Gynecol Reprod Biol.

[7] Steiger RM, Nageotte MP (1990). Effect of uterine contractility and maternal hypotension on prolonged decelerations after bupivacaine epidural anesthesia. Am J Obstet Gynecol.

[8] Clarke VT, Smiley RM, Finster M (1994). Uterine hyperactivity after intrathecal injection of fentanyl for analgesia during labor: a cause of fetal bradycardia?. Anesthesiology.

[9] Van de Velde M, Vercauteren M, Vandermeersch E (2001). Fetal heart rate abnormalities after regional analgesia for labor pain: the effect of intrathecal opioids. Reg Anesth Pain Med.

[10] Grangier L, Martinez de Tejada B, Savoldelli GL, Irion O, Haller G (2020). Adverse side effects and route of administration of opioids in combined spinal-epidural analgesia for labour: a meta-analysis of randomised trials. Int J Obstet Anesth.

[11] Cavens L, Roofthooft E (2022). Neuraxial labor analgesia: is there a place for neuraxial adjuvants beyond opioids. Best Pract Res Clin Anaesthesiol.

[12] Valensise H, Lo Presti D, Tiralongo GM (2016). Foetal heart rate deceleration with combined spinal-epidural analgesia during labour: a maternal haemodynamic cardiac study. J Matern Fetal Neonatal Med.

[13] Damhuis SE, Groen H, Thilaganathan B, Ganzevoort W, Gordijn SJ (2022). Intrapartum epidural analgesia and emergency delivery rates due to fetal compromise by birth weight percentile. Am J Obstet Gynecol.

[14] Macones GA, Hankins GDV, Spong CY, Hauth J, Moore T (2008). The 2008 National Institute of Child Health and Human Development workshop report on electronic fetal monitoring: update on definitions, interpretation, and research guidelines. J Obstet Gynecol Neonatal Nurs.

[15] Gyllencreutz E, Varli IH, Lindqvist PG, Holzmann M (2021). Variable deceleration features and intrapartum fetal acidemia – the role of deceleration area. Eur J Obstet Gynecol Reprod Biol.

[16] Baron RM, Kenny DA (1986). The moderator-mediator variable distinction in social psychological research: conceptual, strategic, and statistical considerations. J Pers Soc Psychol.

[17] Duryea Elaine L, Hawkins Josiah S, McIntire Donald D, Casey Brian M, Leveno Kenneth J (2014). A Revised Birth Weight Reference for the United States. Obstetrics & Gynecology.

[18] (2016). Practice guidelines for obstetric anesthesia: an updated report by the American society of anesthesiologists task force on obstetric anesthesia and the Society for Obstetric Anesthesia and Perinatology. Anesthesiology.

[19] Anim-Somuah M, Smyth RM, Cyna AM, Cuthbert A (2018). Epidural versus non-epidural or no analgesia for pain management in labour. Cochrane Database Syst Rev.

[20] Kotaska AJ, Klein MC, Liston RM (2006). Epidural analgesia associated with low-dose oxytocin augmentation increases cesarean births: a critical look at the external validity of randomized trials. Am J Obstet Gynecol.

[21] Wong CA, Scavone BM, Loffredi M, Wang WY, Peaceman AM, Ganchiff JN (2000). The dose–response of intrathecal sufentanil added to bupivacaine for labor analgesia. Anesthesiology.

[22] Lucovnik M, Blajic I, Verdenik I, Mirkovic T, Stopar Pintaric T (2018). Impact of epidural analgesia on cesarean and operative vaginal delivery rates classified by the Ten Groups Classification System. Int J Obstet Anesth.

[23] Eriksen LM, Nohr EA, Kjaergaard H (2011). Mode of delivery after epidural analgesia in a cohort of low-risk nulliparas. Birth.

[24] Wassen MMLH, Hukkelhoven CWPM, Scheepers HCJ, Smits LJM, Nijhuis JG, Roumen FJ (2014). Epidural analgesia and operative delivery: a ten-year population-based cohort study in the Netherlands. Eur J Obstet Gynecol Reprod Biol.

[25] Bannister-Tyrrell M, Ford JB, Morris JM, Roberts CL (2014). Epidural analgesia in labour and risk of caesarean delivery. Paediatr Perinat Epidemiol.

[26] Fieni S, Di Pasquo E, Formisano D, Basevi V, Perrone E, Ghi T (2022). Epidural analgesia and the risk of operative delivery among women at term: A propensity score matched study. Eur J Obstet Gynecol Reprod Biol.

[27] Sng BL, Leong WL, Zeng Y (2014). Early versus late initiation of epidural analgesia for labour. Cochrane Database Syst Rev.

[28] Clark SL, Belfort MA, Hankins GD, Meyers JA, Houser FM (2007). Variation in the rates of operative delivery in the United States. Am J Obstet Gynecol.

[29] Baicker K, Buckles KS, Chandra A (2006). Geographic variation in the appropriate use of cesarean delivery. Health Aff (Millwood).

[30] Luthy DA, Malmgren JA, Zingheim RW, Leininger CJ (2003). Physician contribution to a cesarean delivery risk model. Am J Obstet Gynecol.

[31] Kreiser D, Katorza E, Seidman DS, Etchin A, Schiff E (2004). The effect of ephedrine on intrapartum fetal heart rate after epidural analgesia. Obstet Gynecol.

[32] Cleary-Goldman J, Negron M, Scott J (2005). Prophylactic ephedrine and combined spinal epidural: maternal blood pressure and fetal heart rate patterns. Obstet Gynecol.

[33] Reddy UM, Weiner SJ, Saade GR (2021). Intrapartum resuscitation interventions for category II fetal heart rate tracings and improvement to category I. Obstet Gynecol.

